# 2540. Can aminoglycoside dosing be improved through use of pharmacokinetic models from other aminoglycosides?

**DOI:** 10.1093/ofid/ofad500.2157

**Published:** 2023-11-27

**Authors:** Jonathan Faldasz, Jasmine Hughes

**Affiliations:** InsightRX, Boston, Massachusetts; InsightRX, Boston, Massachusetts

## Abstract

**Background:**

Model-informed precision dosing (MIPD) has been shown to improve target exposure attainment. However, the clinical effectiveness of MIPD rests on the predictive accuracy of the model used. Despite increased aminoglycoside (AG) use, PK models remain sparse, leading to an ongoing search for viable models. This study aimed to determine whether the use of MIPD incorporating a PK model designed for a single AG drug could improve predictive accuracy in treatment across other AG drugs.

**Methods:**

Patient data entered into the InsightRX Nova MIPD platform during routine clinical care was de-identified and analyzed retrospectively. Patients were included for analysis if they received at least one dose of amikacin (AK), gentamicin (GM) or tobramycin (TO) and at least one therapeutic drug monitoring sample was collected within 48 hours of an administered dose. For each patient and for each model, samples were grouped by dosing interval and iteratively used to predict future levels (Figure 1) using Bayesian forecasting, mimicking clinical implementation of MIPD. Prediction error was assessed using root mean square error (RMSE) and percent of samples within 20% of predicted values.

**Figure:**
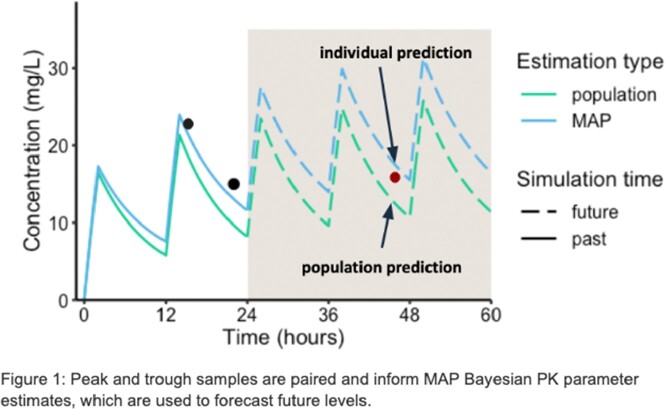

**Results:**

RMSE and percent of samples within range are shown in Figure 2 and Figure 3, respectively. For adults treated with AK (N = 145), the adult models for GM and TO and the pediatric model for GM on average performed better than the drug- and age-matched models. For adults treated with GM (N = 811), the adult AK models performed equally well as the drug- and age-matched models. For children treated with GM (N = 67), the drug- and age-matched model performed the best. For adults treated with TO (N = 239), the AK pediatric model and the adult GM model out-performed the age- and drug-matched model. For children treated with TO (N = 100), all models out-performed the age- and drug-matched model.

**Figure:**
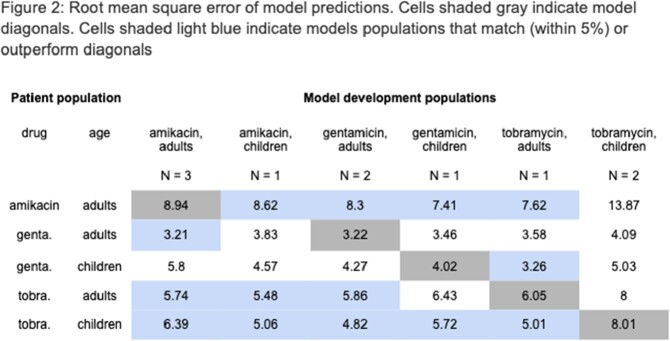

**Figure:**
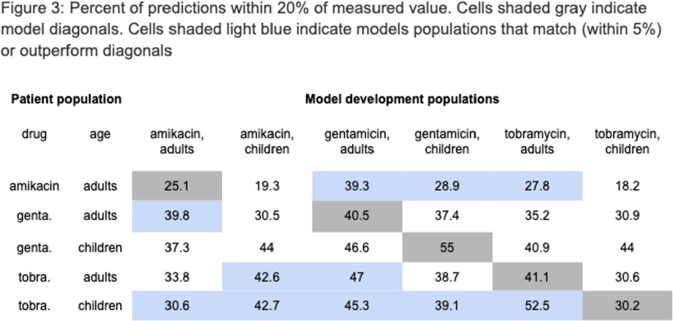

**Conclusion:**

Pharmacokinetic similarity between AGs translates to models that apply well to other drugs within this class, particularly when matching the development population age. However, there is considerable variability between models for a particular drug and age, so individual models should be validated in the intended MIPD population. Further study is needed to test the generalizability of these findings, and explore subpopulations.

**Disclosures:**

**Jonathan Faldasz, PharmD**, InsightRX: Salaried employee **Jasmine Hughes, PhD**, InsightRX: Employee

